# Amorphous silica nanoparticles and the human gut microbiota: a relationship with multiple implications

**DOI:** 10.1186/s12951-024-02305-x

**Published:** 2024-01-30

**Authors:** Massimiliano G. Bianchi, Martina Chiu, Giuseppe Taurino, Enrico Bergamaschi, Francesca Turroni, Leonardo Mancabelli, Giulia Longhi, Marco Ventura, Ovidio Bussolati

**Affiliations:** 1 Lab. of General Pathology, Dept. of Medicine and Surgery, University of Parma, Parma, Italy; 2 Interdepartmental Research Centre “Microbiome Research Hub”, University of Parma, Parma, Italy; 3 Department of Public Health Sciences and Paediatrics, University of Turin, Turin, Italy; 4 Laboratory of Probiogenomics, Department of Chemistry, Life Sciences, and Environmental Sustainability, University of Parma, Parma, Italy

**Keywords:** Amorphous silica nanoparticles, Biocorona, Dysbiosis, Inflammatory bowel disease, Metabolic associated fatty liver disease, Microbiota, SAS

## Abstract

Amorphous silica nanoparticles (ASNP) are among the nanomaterials that are produced in large quantities. ASNP have been present for a long time in several fast-moving consumer products, several of which imply exposure of the gastrointestinal tract, such as toothpastes, food additives, drug excipients, and carriers. Consolidated use and experimental evidence have consistently pointed to the very low acute toxicity and limited absorption of ASNP. However, slow absorption implies prolonged exposure of the intestinal epithelium to ASNP, with documented effects on intestinal permeability and immune gut homeostasis. These effects could explain the hepatic toxicity observed after oral administration of ASNP in animals. More recently, the role of microbiota in these and other ASNP effects has attracted increasing interest in parallel with the recognition of the role of microbiota in a variety of conditions. Although evidence for nanomaterial effects on microbiota is particularly abundant for materials endowed with bactericidal activities, a growing body of recent experimental data indicates that ASNPs also modify microbiota. The implications of these effects are recounted in this contribution, along with a discussion of the more important open issues and recommendations for future research.

**Graphical Abstract:**

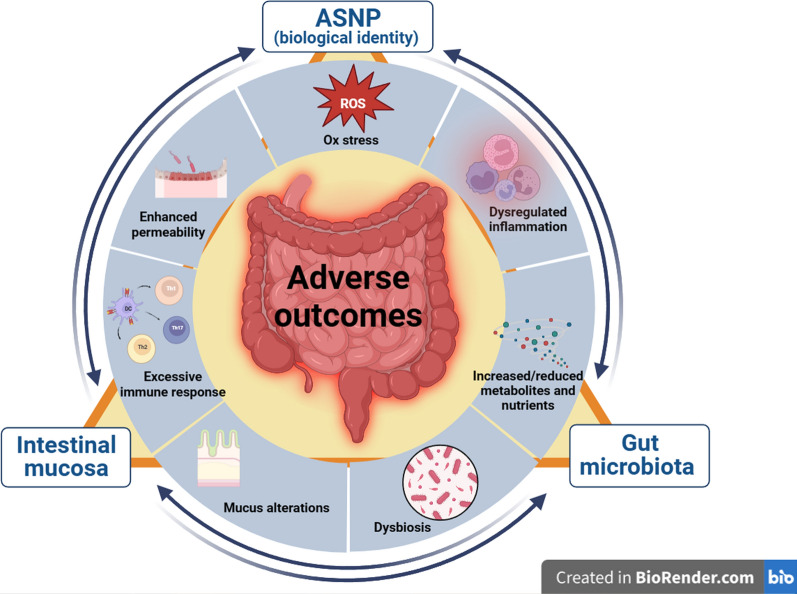

## Introduction

**Fig. 1:**
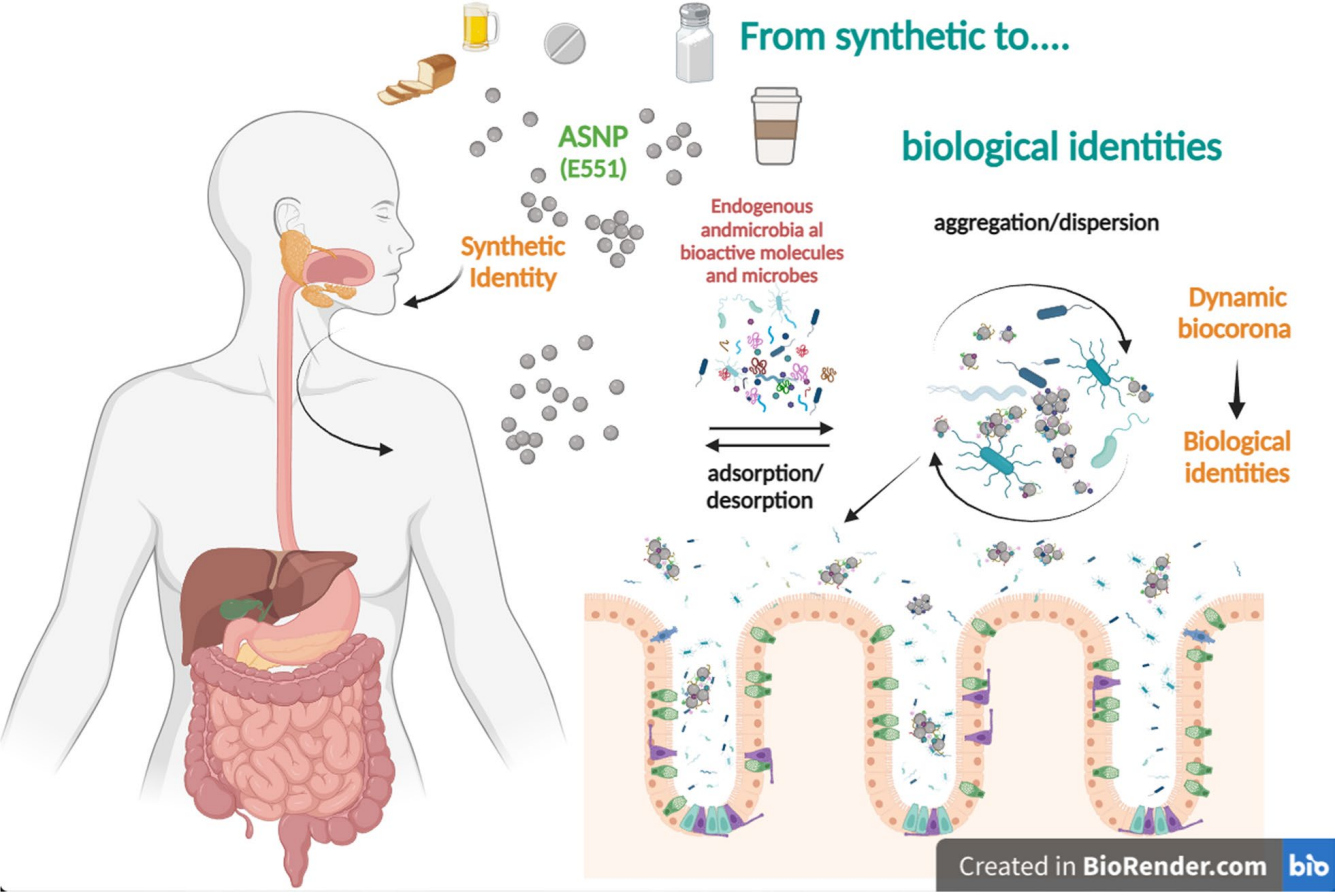
ASNP fate after oral intake. A schematic sketch showing the evolution of ASNP from synthetic (as present in the food matrix) to biological identities (the entities that interact with the gut wall). After ingestion, ASNP are modified by the biological fluids of various gastrointestinal sections, and their surface adsorbs a variety of endogenous, exogenous, and microbial bioactive molecules (e.g. PAMPs (Pathogen-Associated Molecular Patterns acting as macrophage activators), metabolites, nutrients….), forming a dynamic biocorona and providing an array of biological identities that are responsible for the effects. See the text for further explanation

Exploitation as food additives represents a major use for engineered nanomaterials (ENM) in terms of possible impact on human health. While sizable acute toxicity of nanostructured food additives has been repeatedly excluded, their long-term effects are much less characterized. One of the reasons for this incomplete understanding is that nanomaterials undergo a variety of transformations from their ingestion to their elimination or absorption (Fig. 1). This evolving identity is due to the exposure to complex matrices composed of mixtures of organic compounds at different pH and ionic strengths, as well as to the microbial populations specific for the different sections of the gastrointestinal tract.

Silicon dioxide (SiO_2_) nanoparticles are one of the nanomaterials synthesized in large quantities, with a worldwide production of thousands of tons. Synthetic amorphous silica (SAS), used as food additive E551, is exploited for defoaming, chill-proofing, emulsification, viscosity control, anti-settling, and anti-caking [1]. Recently, sugar-bound SAS has also been used as an artificial sweetener to activate tongue taste receptors, providing sufficient sweetening activity with fewer molecules of sugar [2].

Although SAS has been used for several decades as a food additive, the presence of a nanosized fraction (Amorphous Silica NanoParticles, ASNP) has been recognized only in relatively recent years. Food products containing SAS have been shown to contain ASNP up to 43% of the total silica content [3]. Digestion experiments have suggested that dietary ASNP maintain their nanofeatures in the intestine [4]. Previous studies had pointed to no-observed-adverse-effect-level (NOAEL) larger than 1000 mg silica/kg body weight per day [5]. Owing to the lack of obvious acute toxicity, the presence of silica in food is generally considered safe. However, more recent studies have led to a reconsideration of these values [6], and EFSA has concluded that additional data are needed before a definite conclusion is reached about ASNP toxicity [7].

Food-grade ASNP are produced either at high (e.g., fumed or pyrogenic ASNP) or low temperatures (e.g., precipitated ASNP), although the different production processes are not highlighted by a different denomination (both are indicated as E551) or a distinct regulatory discipline. Another form of nanostructured silica, introduced through ingestion, is represented by mesoporous nanoparticles (MSN), usually exploited as drug carriers [8]. For the limited quantities involved they will not be specifically considered in this review unless the mechanism presented is considered of general significance.

However, for widely produced and exploited nanomaterials, such as ASNP, “intentional” ingestion, as food additives or drug carriers, represents only one of the ways of exposure. Unintentional exposure may also occur through water and other environmental contamination [9]. Moreover, exposure to ASNP through routes other than oral administration may lead to contact between the intestine and the nanomaterial, for example if inhaled silica is then ingested or the disposal of introduced nanoparticles involves biliary excretion [10].

Although several reviews concern the impact of ENM on microbial communities, no attempt has been made so far to critically review the literature available on the relationship among ASNP, gut microbiota and the possible consequences of this interaction on human health. This contribution has the purpose to fill this gap, resuming the most relevant data available on the topic and trying to highlight the limitations that have prevented until now the definition of a clear relationship between nanomaterial-dependent dysbiosis and pathological outcomes.

## ASNP fate in the gastrointestinal tract: physico-chemical modifications, bio-corona formation, and the acquisition of diverse biological identities

High specific surface ratio, one of the most characteristic features of nanostructured materials, promotes the adsorption of several substances on the surface of ENM such as ASNP. Therefore, the food matrix in which the particles are mixed is a variable that modulates the fate of ingested ENM [11].

Once ingested, ASNP undergo further changes during passage through the human gastrointestinal tract [10], and their surface characteristics are markedly affected by the changing features of different sections of the digestive system (Fig. 1). For instance, pH undergoes dramatic changes, being around neutrality in the mouth, dropping to 2 in the gastric lumen, rising to 5 in the small intestine, to reach values higher than 7 in the colon. These changes are of paramount importance, influencing both the surface charge and the aggregation state of ASNP. Peters et al. [12] used various food matrices containing E551 or ASNP with an in vitro model mimicking the human digestive tract. The results indicated that NP formed large agglomerates in the stomach but were solubilized again into dispersed NP under intestinal conditions.

In another study, ASNP with a primary particle size below 50 nm were found dispersed in culture media (pH 7.4), where they have a negatively charged surface and moderately aggregate [13]; when dispersed in a gastric-mimetic medium, surface becomes quasi-neutral and ASNP extensively aggregate to re-assume a strongly negative charge and a better dispersed state in a simulated intestinal digestive solution. Changes in surface features, together with the specific composition of the medium, modify the quantity and quality of adsorbed solutes, such as bile salts, microbial metabolites and proteins. The formation of a dynamic biocorona (see Fig. 1) is a characteristic of ENM present in biological fluids and can influence the toxicological properties of the nanoparticles, as extensively shown and reviewed in literature [14–20]. For instance, macrophages and other innate immune cells respond to the biocorona-dependent biological identity of nanomaterials rather than to their synthetic identity [18].

Through modifications of the surface characteristics of ASNP, also the manufacturing process can influence their interaction with gastrointestinal fluids and absorptive processes [21]. Although the sizes of precipitated and fumed ASNP are comparable, their aggregation state and dissolution properties differ. Precipitated ASNP were significantly more absorbed and accumulated in intestinal cells than pyrogenic ASNP. Once penetrated the intestinal cells, ASNP slowly decomposed so that they were no longer identifiable in the bloodstream and kidneys. Pyrogenic ASNP have been extensively characterized during passage through the rat digestive tract [22], demonstrating that, depending on the dose, the physical state of the nanomaterial changes. At medium/high doses, gel-like properties were evident; at low doses, comparable to realistic consumer exposure levels, low gelation occurred, increasing the bioavailability of ASNP.

Different surface characteristics also influence biocorona formation [23]. This different biological behavior has been attributed to the exposure of silanol surface groups which increase the electrostatic interactions of pyrogenic ASNP with the plasma membrane of exposed cells or macromolecules present in the extracellular or intracellular environment. The enhanced chemical reactivity not only perturbs the integrity of the plasma membrane, but it could also involve the generation of hydroxyl radicals, a feature that is largely absent in precipitated ASNP. These features may underlie significant biological effects, such as inflammasome activation [23]. Although specific studies addressing this issue are still lacking, it is possible to speculate that, in the gut context, surface characteristics derived by distinct production processes may lead to differential interactions with fluid macromolecules and the acquisition of novel biological identities.

## Exposure issues: from realistic doses in vivo to in vitro experiments

The contribution by van der Zande et al. [22] indicates that dosimetry, aggregation state, and bioavailability are related. Moreover, based on the scientific literature discussed above, it is highly likely that the effective exposure to ASNP also depends on the dose of E551 ingested. Thus, it is important that the ASNP doses exploited in the experimental models do not markedly exceed the realistic exposure levels expected in real life. From data reported by Dekkers et al. [3], the western consumer intake of silica from food was estimated at 9.4 mg/kg bw/day, of which 1.8 mg/kg bw/day was estimated to be in the nano-size range.

The estimation of realistic exposure levels is important for the correct implementation and interpretation of both in vivo and in vitro experiments. In other words, only experiments performed with realistic dose ranges can yield biologically significant results. Realistic oral exposures are essential to define the level of silicon reached in internal organs after ASNP ingestion in real life and, hence, the possible correlation with pathological changes. Actually, one of the reasons justifying the attribution of low toxicity to ASNP is the low concentration of Si in internal organs upon oral administration of ASNP. For instance, van der Zande et al. [22] did not find any marked increase in Si content in any organ except the spleen upon prolonged oral administration to rats. However, in 2015, a report estimated Si content in human liver at a level comparable to that measured or estimated in animal studies in which adverse effects (e.g., liver fibrosis) were found [24].

The slow absorption of ASNP in vivo is likely attributable to the low permeability of well-differentiated intestinal cell monolayers documented in vitro [25]. However, other, more recent studies, performed with highly sensitive methods, have detected sizable, although modest Si deposition in both the liver and spleen, with a possible pathogenetic role [6, 26]. The extent of the absorption and accumulation is variable. Lee et al. [27] evaluated the absorption of nanostructured and bulk SiO_2_ either in the rat, following single-dose oral administration, or in vitro, using a 3D culture system, consisting of human intestinal follicle-associated epithelium. The absorption of nanoparticles (3.94 ± 0.38%) was greater than that of bulk materials (2.95 ± 0.37%) without significant effects of particle size on in vivo dissolution, biodistribution, or excretion kinetics. Glucose increases oral absorption, possibly due to surface interactions with nanoparticles [27].

Realistic in vivo exposure also provides the rationale for identifying reasonable doses for in vitro experiments. Guo et al. [28], starting from the estimated average exposure of Dekkers et al. [3], considering a total intestinal surface area of 2 × 10^6^ cm^2^ [29], and the wide variation of intake between different subjects [3, 30], selected a range of ASNP doses from high (2 × 10^−3^ mg/ml, 100 × the “physiological” dose), medium (2 × 10^−5^ mg/ml, the “physiological dose”), and low (2 × 10^−7^ mg/ml, 1/100 “physiological” dose) for their in vitro experiments. These doses were lower (in some cases, much lower) than those used in most studies.

The understanding of the consequences of the low absorption of ASNP on distant organs has led to overlook the possible pathogenetic role of the non-absorbed, major aliquot of ingested ASNP that persists in contact with the gut wall. This role may be justified by the interaction of ASNP with intestinal mucosal components or with microbial communities that colonize the gut, two not mutually exclusive alternatives.

Thus, a realistic experimental model of the interaction between ASNP and gut microbiota should consider all these variables, mimicking the conditions in a specific gastrointestinal tract, using realistic doses of ASNP, possibly endowed with the expected biological, rather than synthetic, identity. On the other hand, studies aimed at evaluating the effects of ASNP on gut homeostasis should be performed with models including both host and bacterial cells. However, most of the in vitro studies performed to date have been performed in the absence of bacteria. Therefore, we will first recount the data available on ASNP effects on host intestinal cells or tissues, distinguishing epithelial and immune cells, and then the available evidence on the interaction between ASNP and microbiota.

## Local intestinal effects: genotoxicity, cytotoxicity, inflammation, functional perturbation

ASNP have been reported to induce genotoxicity both in vivo and in vitro [31]. In this systematic review the authors collected the most relevant investigations describing different mechanisms of genotoxicity of ASNP, distinguishing primary effects from secondary effects, due, for example, to reactive oxygen species (ROS) production or inflammation. However, the collected evidence for gastrointestinal effects is limited and, as far as in vitro studies are concerned, the doses exploited are very high. Moreover, even in studies exploiting oral gavage as the administration route, effects on distant organs rather than intestine have been investigated [32–34].

The simplest, yet largely used, in vitro intestinal models for the investigation of adverse effects of ASNP are monolayers of polarized colon-derived epithelial cell lines. Sergent et al. [35] studied the cytotoxicity and genotoxicity of ASNP in the HT29 human intestinal cell line, a mucus-producing model, demonstrating that the nanomaterial produced only slight cytotoxic and genotoxic effects after a sub-chronic exposure of 24 h.

Compared to bidimensional cultures, organoids represent a more physiological model, increasingly adopted in toxicological studies. Exploiting this approach, Park et al. [40], while confirming a very low acute toxicity in vivo, reported sizable toxicity in two-dimensional CCD-18Co cells (described as normal human colon fibroblasts), three-dimensional CCD-18Co spheroids, and human colon organoids, with IC_50_ values of 0.6, 0.8 and 0.3 mM for SiO_2_, corresponding to the high doses of 36, 48, and 18 mg/ml, respectively. Interestingly, the study reached quite peculiar conclusions, with ASNP exerting more powerful toxic effects than TiO_2_ NP (IC_50_ values of 2.5, 1.1 and 12.5 mM in the same models).

Cytotoxicity and genotoxicity can, of course, be linked to a possible role in carcinogenicity. The role of ASNP in chemical carcinogenesis may also be indirect. Indeed, ASNP can adsorb genotoxic agents, increasing their DNA-damaging potential, as proposed in macrophages [36]. In this context, another process of paramount importance is Epithelial-Mesenchymal Transition (EMT), which is linked to malignant characteristics such as invasiveness and metastasis. Setyawati et al. [37] studied the effect of nanoparticles used as food additives on this process exploiting SW480 colorectal cancer cells as in vitro model. Nano-TiO_2_ exposure was clearly linked to EMT through the transforming growth factor-β (TGF-β)/mitogen-activated protein kinase (MAPK) and wingless (Wnt) pathways, but induction of EMT-related changes was also observed with ASNP and hydroxyapatite nanoparticles.

In contrast to HT29 cells, Caco-2 cells, another widely used intestinal model, do not produce mucus and form high-resistance monolayers. A Caco-2 clone, C2BBe1 cells, internalizes ASNP, previously incubated in a simulated intestinal solution, but does not exhibit marked cytotoxicity at the high, but reasonable dose of 10 µg/cm² [13]. Contado et al. and Setyawati et al. also reported a substantial lack of acute toxicity of food-grade ASNP [38, 39]. However, another study [40] indicated that the cytotoxic effects of ASNP depend on the differentiation status of the cells, with undifferentiated cells being more sensitive. This effect may explain the results obtained by Tarantini et al. [41], who described the size- and concentration-dependent cytotoxic and genotoxic effects of ASNP in Caco-2 cells, linked to cell uptake of the nanomaterial and the consequent oxidative stress. Smaller ASNP also induced increased IL-8 secretion, although at a relatively high dose (32 µg/ml). However, the same group only partially confirmed these results in rats exposed through oral gavage to 5, 10, or 20 mg/kg b.w./day of pyrogenic or precipitated ASNP for three days. Comet assay analysis showed neither significant DNA strand breaks nor oxidative damage in any of the tissues tested, including gut. However, the authors reported a weak increase in the percentage of micronucleated cells observed in the colon of rats exposed to the lowest doses of pyrogenic, but not precipitated ASNP [42]. The weak acute toxicity on differentiated Caco-2 cells was confirmed in a systematic study by Hempt et al. [43], who found no evidence of acute cytotoxicity in monolayers of differentiated Caco-2 cells with ten well-defined “real food-grade SAS,” either precipitated or pyrogenic.

Lack of sizable cytotoxicity suggests that a direct inflammogenic effect of ASNP is unlikely. However, an indirect mechanism for inflammation was suggested by the results reported by Setyawaty et al. [38], who demonstrated that, in contrast to ZnO NP, ASNP do not elicit NF-kB activation or cytokine gene induction. However, the same authors also showed an increase in ROS production in ASNP-treated intestinal cells, but the effect was only observed at very high ASNP doses and was not associated with overt cytotoxicity.

The intestinal mucosal barrier regulates the relationships between the body and the outside world through mechanical, chemical, immune and microbial-dependent mechanisms [44]. The mechanical components of the intestinal barrier are the mucus and the epithelial layer, composed of different cell populations and endowed with properties of selective permeability due to tight junctions (TJs) between cells and a wide array of transporters. The chemical mechanisms are due to the complex mixture of gastric acid, bile, digestive enzymes, and antimicrobial proteins produced by the various sections of the gastrointestinal tract. Immune barrier includes innate and acquired immunity, the former based on macrophages and dendritic cells and the latter consisting in the gut-associated lymphoid tissue (GALT), the largest lymphoid tissue in the body. Through a dynamic relationship with host tissues, microbiota cooperates with the other mechanisms to ensure full functionality to the intestinal barrier.

Most of the available data concern ASNP-induced changes in the mechanical mechanisms of the barrier. In a study aimed to assess changes in rat immune system after ASNP oral administration (daily, in doses ranging from 0.1 to 100 mg/kg of body weight for 92 days), Gmoshinski et al. found no modification of intestinal permeability to protein macromolecules (ovalbumin) [45], suggesting a relative insensitivity of the intestinal barrier to ASNP. This study was consistent with the assumption of low toxicity of ASNP on intestinal structure and function, although the ASNP characterization was incomplete, thus preventing a proper comparison with other studies.

A more complex in vitro model consisted of the co-cultures of Caco-2 and HT29 cells. The Caco-2/HT29 model can be further implemented with a lymphoid cell population (Raji lymphocytes) that differentiates into M cells [46]. Using a Caco-2/HT29 co-culture upon acute (4 h) or chronic (5d) exposure, Guo et al. found that the barrier function was not acutely decreased, although it was compromised upon chronic exposure, in association with the generation of ROS and the initiation of pro-inflammatory signaling [47]. Further studies [48] proposed a mechanism involving the disorganization of ZO-1-dependent tight junctional complexes and actin cytoskeleton disruption, in the absence of changes in the expression of tight junctional proteins, with the mucus layer exhibiting a protective effect. In the same model, the shape of the ASNP was also important, with virus-like NP causing a larger permeation-enhancing effect than spherical silica NP of the same size (∼ 60 nm) [49].

The effects of exposure to ASNP on the intestinal barrier, considered as an interactive structure of epithelial and subepithelial tissues, have been studied by Kasper et al. [50] with an in vitro co-culture model consisting of the intestinal cell line Caco-2 and the microvascular endothelial cell line ISO-HAS-1 on opposite sides of a transwell filter membrane. Mimicking Inflammatory Bowel Disease (IBD), the authors obtained a significant barrier disruption, demonstrated by an increase of the damage-related secreted form of the adhesion molecules ICAM and E-selectin and of the pro-inflammatory cytokine IL-8. Under these conditions, ASNP caused a decrease in exosomes bearing sICAM/sE-selectin, a change that authors interpret as an interference with exosomal trafficking, but that may also indicate a mitigation of inflammatory changes.

Organoids can also be used to produce more complete models of the intestinal epithelium than two-dimensional, traditional cultures [51, 52]. The latter contribution highlights the different behavior of intestinal monolayers with or without M cells when the permeability and biological effects of nano- and microplastics are investigated, suggesting that the presence of this specialized population could be important for evaluating the effects of other nanomaterials. It should also be noted that the increase in permeability caused by exposure to ASNP has been proposed as a device to enhance the absorption of proteins of pharmacological interest [53] (see below).

Other functions of the intestine, such as its absorptive activities, seem to be influenced by ASNP. Kolba et al. [54] adopted the model of *Gallus gallus* (broiler chicken) eggs (intra-amniotic administration) to study the effects of TiO_2_, SiO_2_, and ZnO NP on gut health and function. NP type, dose, and the presence or absence of minerals resulted in altered functions and abundance of intestinal bacterial populations (see below). It has been reported that ASNP, while increased the activity of brush border intestinal alkaline phosphatase, significantly affected Fe, Zn, glucose, and lipid absorption, lowering the expression of nutrient transport proteins, damaging the brush border membrane, and reducing the absorptive surface area [47]. Among the transporters of nutrients affected by ASNP, a defect in the peptide transporter OPT-2/PEP-2 has been described, leading to a defective absorption of di- and tri-peptides in the model organism *Caenorhabditis elegans* [55]. After absorption, the peptides are trapped in intracellular vesicles, causing severe growth defects in the exposed worm.

## Effects on immune cells in the intestine: activation and modulation of response

In addition to epithelial cells, the gastrointestinal mucosa contains an important and complex population of innate and adaptive immune cells, which must survey the 200 m^2^-mucosal surface and establish homeostatic relationships with the normal microbiota, while preventing and counteracting tissue invasion from pathogens or toxic substances. The population of innate immune cells comprises macrophages, dendritic cells, and innate lymphoid cells (ILCs), with a predominance of ILC3 cells, whereas adaptive cells include various subsets of T and B cells [56, 57].

Although mostly performed on cells derived from tissues other than the intestine, available experiments indicate that ASNP are not markedly cytotoxic towards innate immune cells, although, when directly compared, cells of the macrophage lineage are more sensitive than intestinal epithelial cells [36]. Most contributions obtained in murine models indicate that ASNP can exert an activating effect on macrophages [58–61], with pyrogenic ASNP apparently more effective than their precipitated counterparts upon acute exposure [59]. However, in human macrophages, when used at low, non-toxic doses, ASNP, rather than activate macrophages, can rather modulate their response to natural PAMPs, such as LPS, leading to the intracellular sequestration of membrane receptors and, thus, interfering with cytokine secretion and activation-associated metabolic changes [62, 63].

In vitro exposure to ASNP, similarly to fine crystalline silica and TiO_2_ nanoparticles, induce MHC-II, CD80, CD86 in murine dendritic cells (DC) and activate the inflammasome, causing a sizable IL-1β-secretion [64]. Winkler et al. [65] generated immature dendritic cells (DCs) and demonstrated that, while they internalize ASNP without exhibiting cytotoxicity or release of interleukin (IL)-1α or tumor necrosis factor-α, they display maturation markers, induce pro-IL-1β and, subsequently, secrete the mature cytokine. In contrast, no IL-1β secretion occurs upon internalization of TiO_2_ or FePO_4_ nanoparticles. The same authors demonstrate that that endosomal pattern recognition and MyD88 are involved in the effect [4]. The activating effect of ASNP on DCs may involve ATP secretion and the purinergic receptor P2 × 7 [66].

Interestingly, the ability to promote activation of immature DC may imply the breakdown of oral tolerance, the process that avoids excessive immune response to antigens, included those contained in food, and autoantigens [67]. These authors have demonstrated that ASNP (39 nm) increased the levels of ovalbumin (OVA)-specific IgG in OVA-tolerized mice, induced the proliferation of OVA-immunized splenocytes, and increased the expression of OVA-specific IgG1, IgE, and IgG2a, indicating stimulation of both the T_H_1 and T_H_2 lymphocytes. The expression of interferon (IFN)-γ (T_H_1), interleukin (IL)-4 and IL-5 (T_H_2), and IL-17 (T_H_17) was also stimulated in a dose-related manner in splenocytes treated ex vivo with OVA. These results are substantially consistent with what was observed by Feray et al. [68], who exposed human monocyte-derived dendritic cells to pyrogenic ASNP and demonstrated that treated cells upregulated the surface expression of CD86 and CD83 (DC activation markers) and the secretion of CXCL-8 and CXCL-12.

As far as adaptive immunity cells are concerned, a decrease of CD4+ T cells and an increase in Treg cells was observed after 28 and 92 days of silica administration in rats with an evident dose dependence [45]. Counterintuitively, these changes were associated with a marked increase of serum TNFα and a decrease of IL-10, of which intestine is a major source. Interestingly, the NOAEL for this “immunotoxicity” (100 mg/kg/d) was much lower than that calculated for systemic toxic effects (see above). These authors wonder what is the mechanism underlying these effects, given the very low systemic bioavailability of ASNP, and attribute an important role to alteration of the marked local immune response observed in a previous study [69]. In co-cultures of dendritic cells and lymphocytes, pyrogenic ASNP increased the proliferation of T-lymphocytes and the production of IL-9, IL-17 A and F by these cells [68]. The influence of bacteria on these effects has been investigated by Malachin et al. [70], who combined the treatment with ASNP with the incubation in conditioned media of cultures of commensal or pathogenic bacteria. Dose-dependence and arrays of cytokines secreted changed according to the bacteria used to produce the conditioned media, and the effects were mediated by both proteinaceous and non-proteinaceous compounds.

Possible microbiota-mediated effects of NP on immune function have been reviewed by Lamas et al. [71]. Authors conclude that NP may simultaneously influence immune cells and intestinal microbiota composition, although their contribution was particularly focused on NP endowed with clear cut antibacterial properties and only marginally concern ASNP. The issue is discussed more thoroughly below.

## Interaction with microbiota: evidence from studies on microbial communities in the environment and model organisms

One of the first data on the possibility that “inert”, silica-based nanomaterials can affect microbial populations was obtained in 2008 [72], with the demonstration that nanometric glasses with different sodium content modified bacterial growth on bovine dentine disks with adherent *Enterococcus faecalis* cells. Silica NP were instead found relatively ineffective, compared with highly toxic silver NP, on arctic soil microbial community [73].

Evaluating the antibacterial activity of medicinal earths, dating to 16th–18th century, natural clays of the same composition (reference clays) and synthetic clays (natural clays spiked with elements such as B, Al, Ti and Fe), Christidis et al. [74] demonstrated that the activity was likely attributable to a fungal exometabolite (bioxanthracene B), produced by *Talaromyces* sp, a fungus of the family of Trichocomaceae (order Eurotiales), historically associated with *Penicillium*.

Beneficial effects on microbial communities were also recorded. Nanosilica (20–40 nm), originated by green synthesis, significantly enhanced microbial populations by influencing the total biomass content, in terms of both C and N, with larger effects than those observed with microsilica, sodium silicate and silicic acid [75]. More recently, growth promoting effects of ASNP on rhizobacteria, mediated by the induced capacity to sustain environmental stress, have been also reported [76].

Lastly, ASNP are able to modulate the metabolic characteristics of microbial communities [77]. The rhizosphere metabolite profile of *Brassica chinensis* L. plants, sprayed with SiO_2_-NPs every 3 days for 15 days, was altered with significant increases or decreases in the relative abundance of several metabolites in various bacterial and fungal genera.

Model organisms offer unique experimental possibilities and have been used in nanotoxicological studies on ASNP. Pandey et al. [78], studying ASNP uptake in the midgut of *Drosophila melanogaster*, found increased expression of *hsp70* and *hsp22* along with caspase activation, membrane destabilization and mitochondrial membrane potential loss, associated with endocytosis-mediated uptake in the midgut cells, documented with TEM.

*Caenorhabditis elegans* has been used to evaluate the relationship between exposure to ASNP and lifespan [79, 80]. Results indicate that ASNP enter intestinal cells and, through the intestine, reach the reproductive tract, causing premature reproductive aging associated with the increased amounts of ubiquitinylated proteins, a finding that suggests an accumulation of misfolded proteins and, hence, an alteration of proteostasis. Since developmental defects were excluded, Pluskota et al. proposed that silica-nanoparticles induce an age-related degeneration of reproductive organs. The possible role of microbiota in the pro-aging effect of ASNP have been investigated more recently [81]. Through a combination of phenotype screening, omics profiling and functional validation, 16 members of *C. elegans* microbiota were screened. Worms grown with *Chryseobacterium* sp. CHNTR56 MYb120 or *Comamonas* sp. 12022 MYb131, were the most resistant to oxidative chemical stress caused by ASNP. RNAseq analysis of young adult worms, grown with each isolate, revealed the enrichment of cellular detoxification mechanisms, vitamin B6 synthesis, TGF-beta and Wnt signaling pathways. Specifically, vitamin B6, determined both in vitro and in vivo, contributes to the improvement of host fitness and was abundant in the isolates and within worms grown with their combination. High levels of glutamine were also found in combination of isolates, suggesting that several bacterial species are grouped in a crosstalk that promotes the growth of *Comamonas* sp. 12022 MYb131 in vivo and the synthesis of vitamin B6 in the worm gut. All together these observations provide a proof-of-principle demonstration that a complex phenotypic effect of ASNP, mediated by microbiota in the host, can be due to a metabolic signal derived from a mutualistic interaction among different bacterial species.

## Interaction with microbiota: evidence from studies on mammalian models

Recently, some investigative efforts have been placed on the possible microbiota-mediated effects on immune function of nanomaterials, with a specific attention paid on ENM endowed with biocidal activities, which favor intestinal dysbiosis [71]. Such studies highlighted that NPs may influence immune cells and cause a moderate to extensive impact on intestinal microbiota composition, with a recurrent signature that favors pathobiont colonization. As far as ASNP are concerned, as an example of nanomaterial not endowed with marked biocidal properties, it has been proposed that effects on microbiota should be taken into account [71]. These considerations suggest the need to investigate possible pathogenetic mechanisms associated with ASNP intake but not necessarily linked to absorption or to direct toxic effects of the ENM on microbiota. For instance, ASNP may adsorb a variety of microbiota-derived macromolecules, which are potential activators of immune cells (PAMPs), thus providing a rationale for the association between ASNP intake and increasingly observed chronic inflammatory conditions. PAMPs adsorbed on ASNP bio-corona may obtain an enhanced access to the bloodstream promoting the activation of systemic inflammatory responses. Another potential pathogenetic mechanism may depend on ASNP-induced changes in the microbiota metabolism with increase or depletion of absorbable bioactive metabolites.

The possibility that biological effects of nanomaterials may be mediated by alterations in microbiota composition has been proposed several years ago [82] and recently reviewed [83]. The first studies on the possible interaction of ASNP with intestinal microbiota were performed in 2015 in rats [84], where no significant changes in the qualitative and quantitative composition of the intestinal microbiota populations were described. Consistently, no change in α- and β-diversity of gut microbiota after an extensive (12 weeks) treatment with a low dose (3 mg/kg/d) of ASNP has been reported [85]. Overall, relative ineffectiveness of ASNP, compared to other engineered nanomaterials (carbon nanotubes, titanium dioxide, cerium dioxide, zinc oxide, nanosilver), was also indicated in one of the first attempts to review the evidence on the effects of nanomaterials on the gut microbiota [86]. In contrast, enhancement of microbial species richness and diversity has been described in mice exposed to ASNP through oral administration, with an increase of genera *Alistipes*, *Lactobacillus*, *Oscillibacter* and *Prevotella*, and a decrease of *Bacteroides* [87]. More recently, several studies [54, 88–90] have presented clear-cut evidence of microbiota changes associated with the toxic effects (e.g. altered transport and barrier functions, inflammatory changes, decrease in mucus, increase of LPS absorption, hepatotoxicity) observed after ASNP exposure.

In a recent investigation, aimed to compare ASNP with micro and nano TiO_2_ through the assessment of several endpoints, ASNP at high doses increased systemic (IL-1α and C-reactive Protein) and local (TNFα and IL-6) inflammatory markers, without modifying the histology of the intestinal tissue [89]. Overall, the pro-inflammatory markers were lower than those observed with titania. At phylum level, ASNP drastically lowered Verrucomicrobia and Bacteroidetes, while increased Firmicutes. Similar changes were observed with titania, at both family and genus levels. All the materials significantly decreased *Akkermansia*, *Barnesiella* and *Bacteroides* genera. Interestingly, these changes were associated with an increase in intestinal LPS content and seem of pathogenetic relevance. In particular, the reduction of *Barnesiella* could hinder intestinal resistance to pathogenic bacteria [91], while the fall in *Bacteroides* may have negative nutritional effects, hindering polysaccharide metabolism and butyrate production [92]. The finding of *Akkermansia* depletion is somehow counterintuitive, since *A. muciniphila* overgrowth is positively correlated with IBD [93], but it is consistent with the observed decrease in mucus layer thickness (see below, Potential roles in pathology and consequences of exposure of susceptible subjects) and with other reports that attribute beneficial effects to this species (see, for instance [94]).

On the same line of evidence, Diao et al. [95] treated young mice with vehicle or ASNP for 28 days and studied microbiota changes, through 16S ribosomal RNA (rRNA) gene sequencing, and the neurobehavioral functions. ASNP exposure was significantly associated with spatial learning defects, memory impairments and locomotor inhibition but did not trigger evident intestinal or neuronal inflammation. Gut microbial diversity was enhanced in treated mice, with increased Firmicutes and Patescibacteria. Authors claim that the disruption of gut–brain axis may be attributed to specific substances, yet to be identified, able to decrease the expression of both *Vipr1* and *Sstr2* in the gut and in the brain. *Vipr1* encodes for the receptor of vasointestinal peptide 1 while *Sstr2* for a receptor for somatostatin.

No adverse health effects have been instead described by Landsiedel et al. [96] after a 28-day treatment of male Wistar rats with high doses of ASNP (1000 mg/kg body weight/day). However, substantial modifications of the gut microbiota were detected, with an increased abundance of Prevotellaceae and the reduction of several genera. Interestingly, authors associated microbiota studies with plasma metabolomics, finding several metabolites significantly altered in treated animals and, in particular, a decrease (0.78-fold of control levels) in indole-3-acetic acid, a ligand of the arylhydrocarbon receptor (AHR) critical for regulating immunity, xenobiotic metabolism and other important functions.

Administration of mesoporous silica nanoparticles (MSN) in mice is also associated with changes in the gut microbiota composition [97]. After 2 weeks of exposure, serum ALP, ALT, AST and TNF-α levels increased, infiltration of inflammatory cells in the spleen and intestines was detected, and colon epithelial apoptosis was observed. At cell level, mitochondrial membrane potential decreased and NLRP3 inflammasome expression was stimulated, along with that of TLR4 and p-NFκB, while the autophagy-related proteins LC3-II and Beclin1 were repressed. MSN significantly changed the intestinal microbiota diversity: the relative abundances of Firmicutes, Actinobacteria and Proteobacteria significantly increased, while Verrucomicrobia markedly decreased. The Firmicutes/Bacteroidetes (F/B) ratio, a marker of the overall state of intestinal microbiota [98, 99], increased in a dose-dependent way. Moreover, these authors presented a metabolomic analysis of intestinal content, which indicated a deviation from normal pattern in NP-treated animals. In animals treated with the highest dose of ASNP, the levels of 36 metabolites were increased and the levels of 5 metabolites were significantly decreased. The biosynthesis of phenylalanine, tyrosine and tryptophan, together with protein digestion and absorption, were the major pathways involved. These changes were significantly correlated to modifications in the abundance of specific bacterial genera.

Changes in the gut microbiota composition were detected also by Bredeck et al. [100] with two distinct protocols. In mice receiving food containing 1% SiO_2_ NP for three weeks the relative abundance of Actinobacteria was increased in the absence of changes in α- or β-diversity. On the other hand, a contribution aimed at evaluating the effect of different preparations of MSN for drug delivery indicated that short term exposure of rats to the ENM caused a decrease in Verrucomicrobia and an increase in *Candidatus saccharibacteria*. Interestingly, only the single type of MSN that perturbed microbiota (MCM-41) was associated to inflammatory changes in the colon mucosa [90]. Perturbation of the gut microbiota composition by ASNP was also observed in male C57BL/6JRj mice exposed to dietary NPs mixed at doses relevant for human exposure (0.8, 8 and 80 mg/kg pellet). After 24 weeks, no evident toxicity was recorded but the β-diversity was dose-dependently disrupted, along with a decrease of the bacterial derived short chain fatty acids. These effects were substantially reversible [101].

The mechanisms underlying the reported effects of ASNP on microbiota diversity are still obscure. The alteration of the microbiota composition described after ASNP intake could be partially promoted by a modified biodistribution of nutrients essential to microbiota maintenance and adsorbable in the ASNP bio-corona. This hypothesis, although not yet validated, may represent a suggestive field of investigation aimed to clarify a possible correlation between ASNP-associated nutritional availability and gut dysbiosis.

In turn, changes in microbiota diversity can be associated with possible alterations of inflammatory signaling and immune response in the host. Although not directly related to intestinal microbiota, other recent studies provide a proof-of-principle experimental evidence that ASNP can modulate the functional outcomes of the metabolic interaction of bacteria with innate immune cells [70]. Interestingly, these studies were based on human dendritic cells treated with ASNP in the presence of conditioned media (CM) from cultures of commensal or pathogenic bacteria. ASNP dose-dependently modified the array of cytokines produced, which also varied according to the bacteria used to produce CM. A preliminary analysis revealed that both proteinaceous and non-proteinaceous CM components were involved.

Microbial products can be sensed and recognized non only by innate immune cells but also by other cell populations of the intestinal mucosa. Intestinal epithelial cells are indeed endowed with a large array of PRR receptor [102], each of which characterized by specific distribution and regulation. For instance, TLR4 expression is time dependent, with higher levels in fetal intestine, and mainly localized in the crypts [103]. Moreover, the expression was greatly increased in neoplastic compared with normal colonocytes [104, 105]. On the other hand, TLR2 was more expressed in colonocytes from obese than from lean subjects [106].

The metabolic interaction between host cells and microbiota components can be complex and biunivocal. Xu et al. [107] studied the metabolic relationship between colon carcinogenesis and Gram-negative bacteria. Through the TLR4/MYD88 pathway, Gram negative bacteria promote the induction of Delta-5 desaturase (FADS1), a rate-limiting enzyme for arachidonic acid synthesis from linoleic acid, which is upregulated in colorectal cancer. The increased availability of this polyunsaturated acid promotes cancer growth enhancing PGE2 synthesis. Depletion of Gram-negative bacteria through specific or broad-spectrum antibiotics abolishes the effect. Therefore, any marked changes of microbiota composition, potentially due to ASNP intake, may have heavy effects on colon homeostasis through the establishment of metabolic cross talks involving the activation of inflammatory, TLR-dependent pathways.

## Potential roles in pathology and consequences of exposure of susceptible subjects

The pathogenetic implications of oral exposure to ASNP should be investigated considering not only the effects of the nanomaterial on the intestinal tissue and the various cell populations involved, but also the effects on the microbiota, keeping in mind that, in turn, intestinal tissues and microbiota are also able to influence each other.

**Fig. 2:**
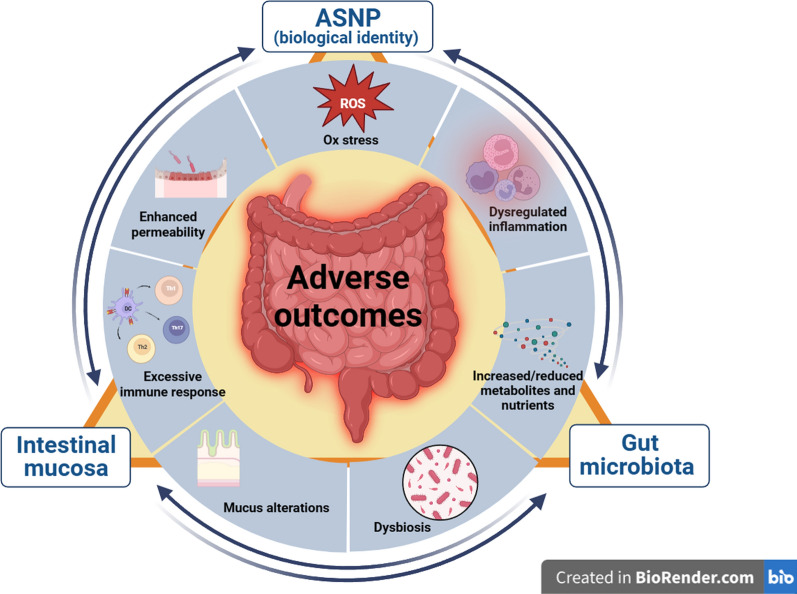
Pathogenetic relevance of the trilateral relationship among ASNP, intestinal mucosa and microbiota. Adverse outcomes (here focused on the intestine but potentially involving other organs) derive from a relatively limited number of main pathogenetic mechanisms activated by the interactions among ASNP, microbiota and intestinal mucosa. Each of these can influence the other two. Depending on their biological identities, ASNP can modify microbiota and damage intestinal mucosa (see text). In turn, through the secretion of products and metabolites, gut cells and microbiota can modify the biological identity of ASNP and, hence, their biological effects. The bidirectional interaction between microbiota and the intestinal mucosa is documented by a rich literature

The complex trilateral interactions that can be envisaged (Fig. 2) are not yet validated by experimental evidence but open the pathway to potential research lines on several conditions. Among the various possibilities, two are worthy of peculiar consideration.

**Fig. 3:**
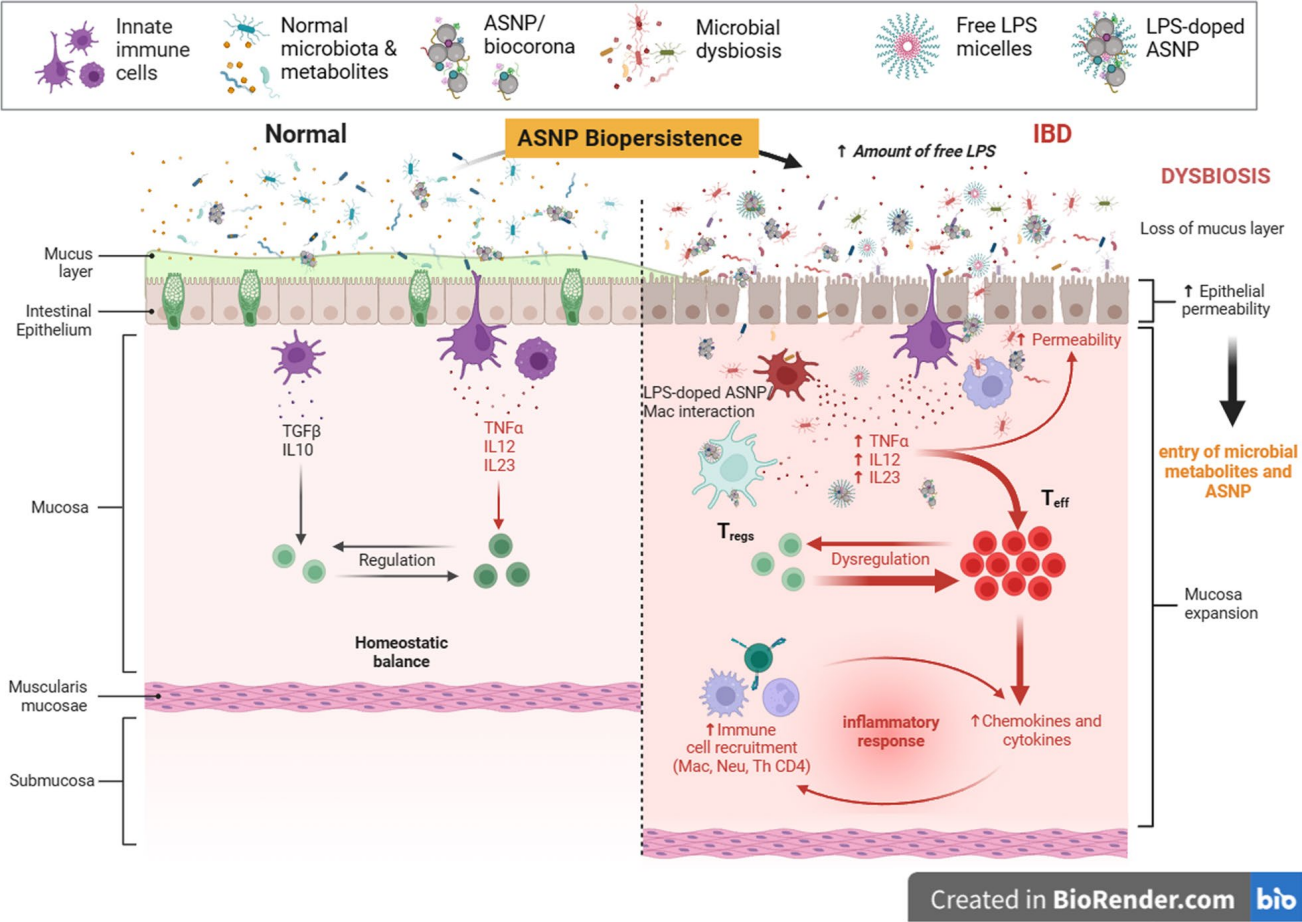
Putative mechanisms involved in microbiota-mediated ASNP contribution to IBD. Once reached intestinal mucosa, biologically modified ASNP may persist into the mucus layer interacting with the gut microbiota. ASNP-microbial interaction may shift microbial diversity through possible different mechanisms (see text), such as nutritional advantages for selected bacteria due to the delivery of adsorbed metabolites. The resulting dysbiosis is associated with a reduction of mucus secretion, increased amounts of selective LPS variants, alteration of epithelial barrier function, and penetration of inflammatory agents (such as LPS-doped-ASNP). Mucosal innate immune cells (dendritic cells, neutrophils and macrophages) engulf components of the altered microbiota with activation of the inflammatory response and the consequent recruitment of selected T-lymphocyte subsets

In the case of IBD (Fig. 3), the literature about the pathogenetic role of microbiota alterations is rich and consolidated, and therapeutic approaches based on this relationship have been already proposed (see the recent reviews [108–111]). Much less is known on the possible influence of ASNP on the development of IBD. However, Ogawa et al. [85] have demonstrated that the daily intake of 10-nm (but not of 30-nm) ASNP for 12d exacerbates dextran sulfate-induced experimental colitis in wild-type C57BL/6J mice. The exacerbation was not observed in mice deficient of the ASC inflammasome, pointing to this complex as the ASNP target. The exposure period seems of paramount importance in determining the toxicological outcome. Indeed, Cabellos et al. [112] found no significant toxic effects either locally (intestine) or in distant organs after a 5d administration of high doses of non-porous or mesoporous ASNP. On the contrary, after a 28d-treatment, the cited contribution by Yan et al. [89] documented inflammatory damage to the intestine, although ASNP had weaker effects than nano-TiO_2_. Changes in the microbiota composition were recorded (see above) in mucus-associated bacteria, such as *Barnesiella*, *Bacteroides* and *Akkermansia*, which were found markedly decreased. This observation is important since the same contribution demonstrated a decrease of MUC2 expression, and a significantly reduced thickness of intestinal mucus in ASNP treated mice. Thinning of mucus layer can contribute to intestinal damage since it favors the direct contact of bacteria with intestinal epithelial cells, lipopolysaccharide absorption and TLR4-dependent inflammatory activation. Mucus production is an example of intestinal cell function subjected to the integrated control by host and microbiota components. Indeed, co-cultures of human intestinal cells and microbiota components promote the expression of mucin genes in the host cells and of mucus-metabolizing enzymes in bacterial populations [113].

Quite paradoxically, alterations in intestinal barrier associated to ENM ingestion (see above) have been also exploited as a convenient device to increase oral drug absorption (reviewed in [114]). In this context, ASNP have been used as drug carriers for intestinal inflammatory diseases in experimental models since several years (see, for instance, [115–121]). Compared to free drugs, compounds carried by ASNP exhibit lowered toxicity and a tendency to accumulate in inflamed tissues, which renders them particularly interesting in IBD [115]. However, the possibility that, in susceptible subjects like IBD patients, even the low doses of mesoporous silica used may have adverse effects on microbiota has not been investigated yet.

Some studies have explicitly targeted or detected microbiota alterations as therapeutic mechanisms or outcomes. Cheng et al. [122] have synthesized multilayer-coated mesoporous silica to release drugs (e.g. hydrocortisone) specifically in the colon. These nanomaterials are stable in acidic (e.g. stomach) and neutral (e.g. intestine) environments but release their cargo in the presence of azoreductase produced by the colon microbiota (mimicked in vitro by dithionite). In vivo efficacy was confirmed in the dextran-sulfate induced colitic mouse, where the preparation favored epithelial barrier integrity and elevated the levels of AHR agonists, derived from the restored metabolism of tryptophan. Moreover, lowered levels of inflammatory cytokines and a partial restoration of colitis-induced dysbiosis was observed, pointing again to a strict relationship between microbiota alterations and mucosal inflammation.

Yin et al. [123] developed a mesoporous silica nanoparticle conjugated with long-chain fatty acids and covered with enteric coating. The NP were absorbed and transported via the mesenteric lymphatic system, which is known to be altered in IBD. The in vivo efficacy was evaluated in the IL-10 KO experimental colitis mouse, where laquinimod-loaded nanoparticles were compared to the free drug and found more effective. Lymphangitis was suppressed and lymphatic drainage restored, with lymphangiogenesis inhibition. As in the study of Chen et al., also the alterations of gut microbiota associated with experimental colitis were reversed.

The other condition is represented by Non-Alcoholic Fatty Liver disease (NAFLD, now defined as Metabolic Associated Fatty Liver Disease, MAFLD), one of the most common liver disorders in Western world. MAFLD is characterized by hepatic steatosis, which can proceed, not necessarily, to hepatocyte death, inflammation, fibrosis and, eventually, liver cirrhosis and hepatocellular carcinoma. This condition has a multifactorial nature, and no specific pharmacological treatment is available yet.

Recent contributions demonstrate that food-grade silica nanoparticles may cause steatogenic changes in liver through a wide alteration of gene expression even at doses compatible with human exposure [124]. Evidence for hepatotoxicity of nanosilica was also reported in the past, although not directly linked to MAFLD. Prolonged oral administration of high doses of pyrogenic ASNP was associated with an increased incidence of liver fibrosis, accompanied by a moderate, but significant increase in the expression of fibrosis-related genes in liver samples [22]. Hepatotoxicity was also observed by Medina-Reyes et al. [88], together with gastrotoxicity and, more importantly, alterations in gut microbiota, changes attributed to oxidative stress as the main mechanism. Interestingly, ASNP hepatotoxicity is greatly increased in the mouse model of metabolic syndrome induced by fructose [33]. In this model, ASNP exposure, while improving insulin resistance, greatly enhanced liver inflammation and fibrosis in metabolic syndrome mice.

**Fig. 4:**
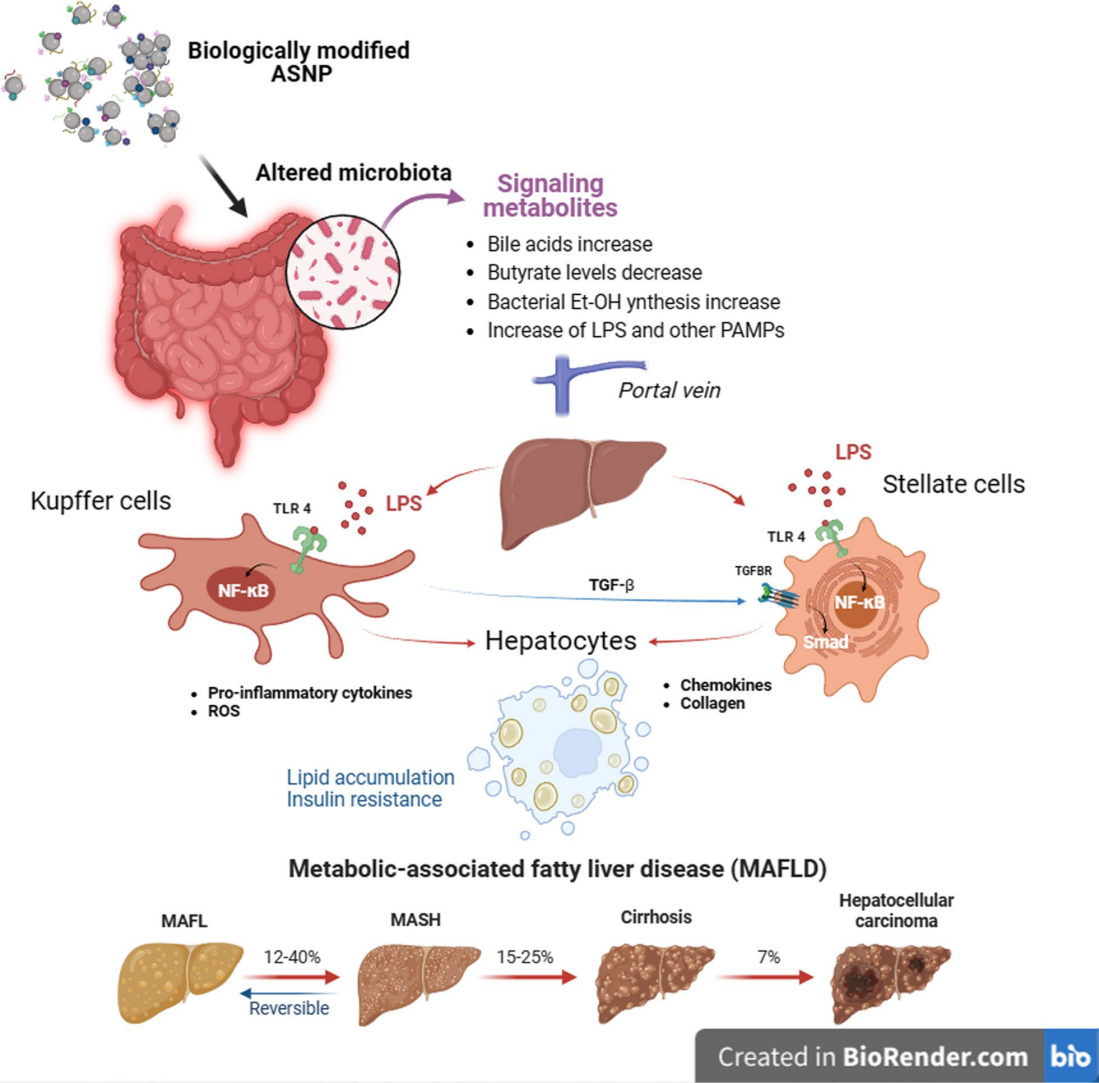
Putative mechanisms for a role of microbiota-mediated ASNP effect on MAFLD. The adsorption of exogenous and endogenous bioactive molecules to ASNP may change the microbial gut signature leading to dysbiosis and rewiring of microbiota metabolism [e.g. reduction of butyrate). The alteration of homeostasis may impair epithelial barrier function causing increased portal blood levels of endotoxin and other PAMPS. Resident liver Kupffer and stellate cells, activated through TLR4-dependent signaling, secrete both pro-inflammatory and fibrogenic cytokines. The establishment of a chronic inflammatory condition may account for the alteration of glucose metabolism in hepatocytes (insulin resistance) and the accumulation of lipids (metabolic-associated fatty liver (MAFL)] and favors the evolution toward metabolic-associated steatohepatitis (MASH)

On the other hand, the relationship of microbiota alterations with metabolic syndrome, MAFLD and liver fibrosis are well known [125–127] and, most recently, mechanistic data have been produced in vitro, which link fibrotic changes to trimethylamine N-oxide (TMAO), a bacterial metabolite [128] associated with diabetes and obesity through epigenetic mechanisms [129, 130]. Interestingly, a recent contribution proposes that some of the hepatic alterations due to exposure to an exogenous, poorly adsorbed material, like microplastics, can be attributed to changes of the microbiota, rather than to direct effects of the material itself on the liver [131], suggesting that this can be possible for other substances. Thus, ASNP may favor MAFLD (a) directly, (b) through changes in intestinal barrier permeability, promoting the increased exposure of innate immune cells and stellate cells in the liver to bacteria or bacterial products, or (c) causing a dysbiosis that may in turn contribute to the changes reported in (b) or exert a steatogenic activity through the increase or decrease of specific metabolites (Fig. 4).

In addition, alterations in intestinal barrier associated with exposure to ASNP (see above) have been exploited as a convenient device to increase oral drug absorption and efficacy for MAFLD. Jin et al. [132] used hollow MSN with encapsulated ammonia borane that produced a sustained H_2_ release in the gut. Upon several weeks of treatment, the nanomaterials counteracted diet-induced (in C57BL/6 N mice) and genetic mutation-induced (in *db/db* mice) early-stage MAFLD, along with obesity and diabetes, without apparent toxicity. Authors report microbiota changes with, among others, increased abundance of *A. muciniphila*. However, while extensive antibiotic treatment prevented MSN protective effects on glycemic control, it did not suppress the effects on lipid accumulation in the liver, casting doubts on the microbiota role in this particular model.

Although MAFLD and IBD certainly represent the most promising fields of investigation, the trilateral interaction described in Fig. 2 may account for other potentially pathogenetic effects of ASNP. In the contribution by Perez et al. cited above [101], the sub-chronic administration of ASNP, altering the microbiota diversity and the production of short-chain fatty acids, was associated with a decrease in the liver expression of IL-6, circulating triglycerides and urea nitrogen, leading to possible implications for the well-known relationships between microbiota, increased inflammatory tone and metabolic disease (see the recent review by Klag et al. [133]).

The potential involvement of ASNP in common diseases elicit the possibility that their effects may be more important in specific categories of susceptible subjects. Preliminary evidence may support this hypothesis. In mice, rendered immunodeficient with the antitumor alkylating agent cyclophosphamide, a subchronic (12d) exposure to ASNP significantly increased the abundance of *Lactobacillus*, *Sphyngomonas*, *Sutterella*, *Akkermansia*, and *Prevotella*, and lowered *Ruminococcus* and *Allobaculum* [134]. No evident difference was detected in immune functions after this relatively short treatment and, unfortunately, it was not assessed if the treatment was able to produce microbiota changes also in control, immunocompetent animals (where other studies have instead documented a decreased abundance of *Akkermansia*, see above). However, it has been known since several years that microbiota changes are involved in the adverse effects of chemotherapy [135], and the relatively short period of treatment may have prevented the possibility of detecting more evident effects of the reported dysbiosis.

## Conclusions: open issues and future research lines

The evidence recounted in this contribution already allows to reach some conclusions, although the field is rapidly evolving. Available data suggest that, after oral ingestion of ASNP, intestinal mucosa, in its major components of epithelial and immune cells, is indeed exposed to a significant portion of nanoparticles. Thus, since the fraction of the ASNP absorbed is limited, the majority of ASNP tend to persist in contact with the intestinal wall, potentially causing local effects.

Moreover, although devoid of a significant bactericidal activity, as well as of a clear-cut acute cytotoxicity for human cells, ASNP can effectively modify mammalian gut microbiota. Some of the changes observed have been repeatedly involved in increased risk for several conditions such as IBD. Other changes, affect *A. muciniphila*, a known modifier of the mucus layer that covers intestinal epithelium associated with beneficial effect on host health.

As a consequence, ingested ASNP can affect extra-intestinal tissues through at least three ways: (i) through direct effects of the small fraction of absorbed ASNP; (ii) through indirect effects, mediated by the local activity of ASNP on epithelial and immune cells of the intestinal wall; (iii) through indirect effects mediated by changes of microbiota composition and/or changes mediated by microbiota metabolism.

These considerations raise several issues. First, given the evolving biological identity that ASNP assume during their passage through the gastrointestinal tract, effects on microbiota, as well as those on the intestinal and other tissues, may be attributed to either ASNP or components of the bio-corona. Adsorption to nanoparticles may modulate (usually enhancing) the biological activities of bioactive molecules, such as bacterial lipopolysaccharide, as known for ASNP and other nanoparticles [59, 136]. Thus, the presence of nanoparticles, even of nanoparticles devoid of intrinsic activity, may have biological effects enhancing or modulating those of substances present in the intestinal lumen.

Second, information about microbiota variability in normal subjects is still incomplete. Moreover, most of the scientific literature available, and recounted above, has been obtained in rodent models. Thus, the possibility that ASNP effects may be different depending on the individual microbiota characteristics is likely.

Third, on the same line of reasoning, also reduced microbial biodiversity associated to human conditions may be more susceptible to changes induced by exposure to ASNP.

Fourth, changes in microbiota composition are obviously associated to changes in the production of metabolites and bio-active compounds present in the intestinal content and potentially absorbable. It is likely that metabolomic changes underlie most of the effects attributed to microbiota in human pathology. Thus, the characterization of these changes, and the identification of the responsible compounds is of paramount importance.

These issues will need intense research activity, both in vivo and in vitro, to achieve mechanistic information beyond epidemiological evidence and genetic characterization of microbial diversity and abundance. To this purpose, the reproduction of normal or pathologic microbiota in bio-reactors and advanced culture models will constitute important devices to assess the effect of exposure to ASNP under experimentally controlled conditions mimicking real life exposure.
